# Supplementary material for: Evaluation of the Hematological and Serum Biochemistry Parameters in the Pre-Symptomatic and Symptomatic Stages of ALS Disease to Support Early Diagnosis and Prognosis
Source: Cells. 2022 Nov 11;11(22):3569. doi: 10.3390/cells11223569 (PMC9688239; doi:10.3390/cells11223569)
Supplement: Supplementary file 1 [file cells-11-03569-s001.zip › cells-2019452-supplementary.pdf]

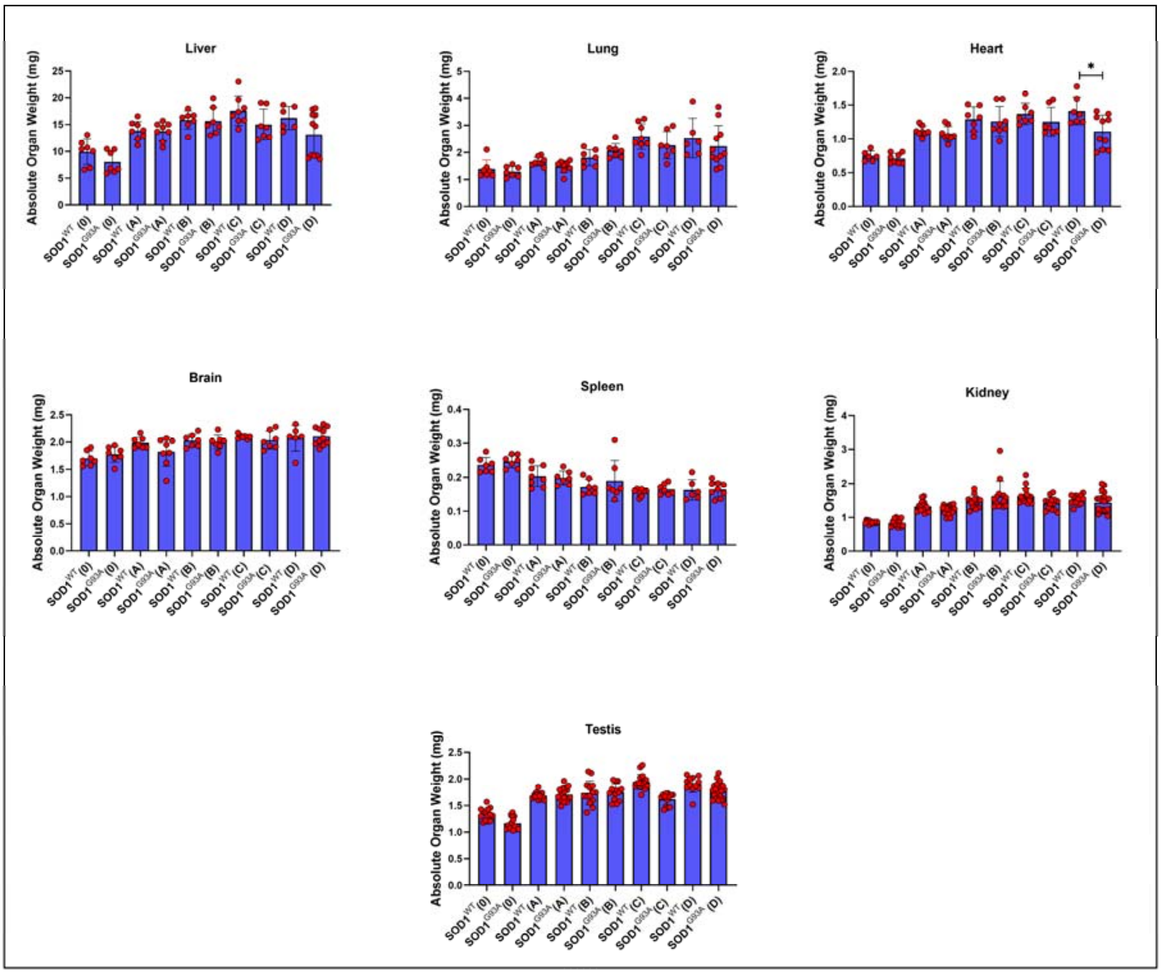

Figure S1: Absolute weights of the SOD1^{G93A} and SOD1^{WT} rats. All results were given as mean ± SD of n = 6–8 animals for each group. Notes: * $p \leqslant 0.05$.
